# An Analysis of Citizen Science Based Research: Usage and Publication Patterns

**DOI:** 10.1371/journal.pone.0143687

**Published:** 2015-11-23

**Authors:** Ria Follett, Vladimir Strezov

**Affiliations:** Department of Environmental Sciences, Macquarie University, North Ryde, NSW, Australia; University of Bologna, ITALY

## Abstract

The use of citizen science for scientific discovery relies on the acceptance of this method by the scientific community. Using the Web of Science and Scopus as the source of peer reviewed articles, an analysis of all published articles on “citizen science” confirmed its growth, and found that significant research on methodology and validation techniques preceded the rapid rise of the publications on research outcomes based on citizen science methods. Of considerable interest is the growing number of studies relying on the re-use of collected datasets from past citizen science research projects, which used data from either individual or multiple citizen science projects for new discoveries, such as for climate change research. The extent to which citizen science has been used in scientific discovery demonstrates its importance as a research approach. This broad analysis of peer reviewed papers on citizen science, that included not only citizen science projects, but the theory and methods developed to underpin the research, highlights the breadth and depth of the citizen science approach and encourages cross-fertilization between the different disciplines.

## Introduction

Public involvement in scientific discovery can be tracked through recorded history [1] with the earliest records dating back 1,910 years for locust outbreaks in China [2]. Recently there has been a significant increase in public involvement in scientific research, now referred to as “Citizen Science”. Alan Irwin [3] was one of the first to use the term “Citizen Science” in 1994 in the context of describing expertise by lay people. This term was soon modified to describe a research technique using members of the public to gather or analyse scientific data [4]. Citizen science is defined by the European Commission Green Paper as “general public engagement in scientific research activities where citizens actively contribute to science either with their intellectual effort, or surrounding knowledge, or their tools and resources” [5].

Citizen science engages the public in scientific projects that are difficult to conduct solely by scientists who lack the resources to gather or analyse data on a large scale [6]. Citizen science engages interested volunteers in a wide variety of projects including monitoring wildlife [7, 8] and the environment [9], as well as classifying images [10–12], transcribing old records [11, 13] and annotating images from past biodiversity collections [14]. Project objectives range from supporting scientific investigations within academic institutions to increasing the interest and knowledge of the general population on science [15].

Citizen science projects can be classified in several different ways. An initial classification for these projects was based on the type of volunteer involvement, dividing them into [4, 12, 13]:

- Contributory, where the participants contribute to data collection, and sometimes help analyse the data and disseminate results.
- Collaborative, where citizens also analyse samples, data and sometimes help design the study, interpret the data, draw conclusion and disseminate the results.
- Co-created, where citizens participate at all stages of the project, including defining the questions, developing the hypotheses, right through to discussion of the results and answering new questions.

An alternative classification for specific citizen science projects has been suggested by Wiggins and Crowston [16] that is based on the goals of the study and identified the following five mutually exclusive and exhaustive types of projects:

- Action projects are initiated by volunteers designed to encourage intervention in local concerns such as improving water quality in their local stream [17].
- Conservation projects address natural resource management goals, for example a project to monitor the type and abundance of beach litter [18].
- Investigation projects focus on scientific research goals in a physical setting, for example a detailed study of otter demographics in California [19].
- Virtual projects also focus on scientific research goals, but are entirely based on information technology with all volunteer interaction occurring on-line such as in Galaxy Zoo, where volunteers find and classify galaxies [20].
- Education projects that are often performed in the classroom or school grounds as part of the science curriculum, for example a butterflies and ground squirrel monitoring study [21].

An additional way of classifying citizen science projects was based on the topic being studied, for example astronomy, archaeology and biology [16].

Research into the citizen science method which underpin the citizen science projects included research into the theory of citizen science [22, 23] and methods applicable to citizen science projects [24, 25] as well as validation techniques [26, 27], studies on motivating volunteers [28, 29] and more general review and overview articles [30, 31].

It has been recently argued that Citizen Science has emerged as a distinct field of inquiry, covering not only citizen science projects but the discipline of citizen science itself [23]. Neylon and Wu [32] state that the most important means of science communication today is through scientific publications. An analysis of peer reviewed papers demonstrate growth of citizen science in scientific literature and the areas to which citizen science was applied. Both Web of Science and Scopus are databases used for searching peer-reviewed articles. Google Scholar is also a database that contains peer-reviewed articles, but also contains non-peer-reviewed publications, or “popular scientific literature and unpublished reports or teaching supporting materials” as termed by Aquillo [33]. For this reason an analysis of articles in the Web of Science and Scopus databases is a recommended baseline for search of published peer-reviewed articles, although, in the case of citizen science publications, the true extent of these publications would be larger as many other studies are published in non-peer-reviewed literature sources and would not be referenced in these two databases [34].

The aim of this work was to highlight the broad landscape of citizen science by monitoring the use of the term “citizen science” in peer-reviewed published papers listed within the Web of Science and Scopus databases. This approach not only highlighted to diversity of research projects using citizen science and the changes that had occurred over time, but also the significant increase in research into methods that underpin citizen science, which will encourage the future application of citizen science in the scientific output.

## Methods

This research is based on a review of peer-reviewed articles collected from the Web of Science [35] and Scopus [36]. All articles with “citizen science” in the topic were extracted into csv files for years up to and including 2014 using the export tools available within Web of Science (S1 File) and Scopus (S2 File) and combined into a single list. The information included in the combined list were the name of authors, title of the article, the source title, the abstract and the year of publication. Google scholar is another source of articles but was not included in this analysis as this source includes both peer-reviewed and unreviewed articles such as technical reports and drafts [37].

**Fig 1:**
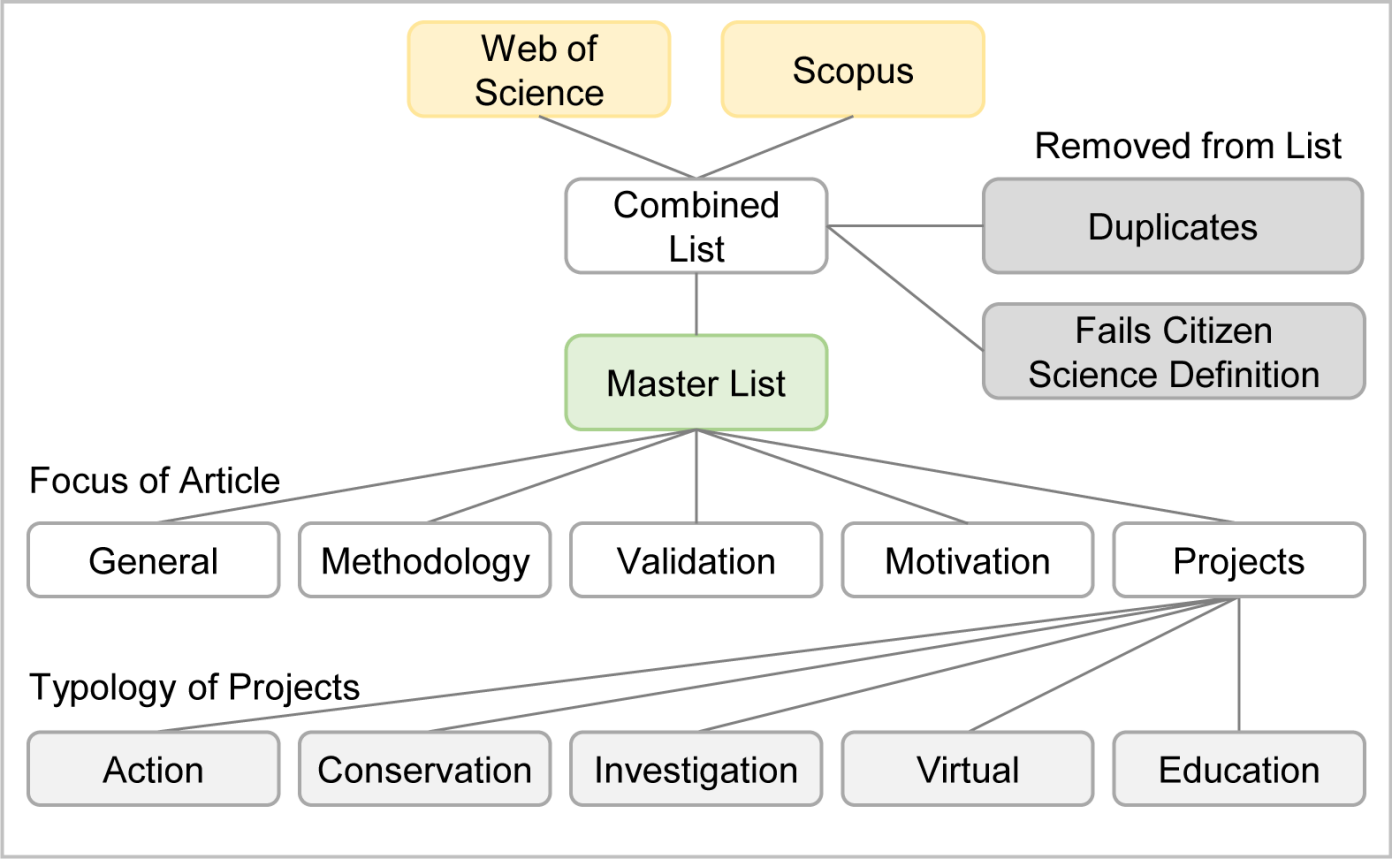
Classifying extracted publications into various categories.

The second step (Fig 1) was to ensure that the combined list of references contained no duplicates and were on the subject of citizen science as defined by the European Commission Green Paper [5]. Articles with the same title and authors were considered duplicates and excluded from further analysis. The title and abstracts of these articles were examined, and articles not satisfying the citizen science definition, such as crowdsourced funding, surveying citizens to provide input for research, science education to citizens, political science and government, citizen’s jury, science tools useful for citizens and tweeting science information to citizens were excluded from further analysis. The resulting list became the master list for analysis.

The third step was to examine the titles and abstracts of papers in the master list produced above to determine which papers described specific citizen science projects. The papers outside the projects category described citizen science more generally, and aspects of citizen science, such as the theory, methodology, validation techniques and the benefits to the participants. In this work, the publications outside the project category were classified into: (i) articles investigating and proposing citizen science methodology or discussing the theory of citizen science, (ii) articles investigating and proposing validation techniques, (iii) articles exploring the motivation of participants and the effects of participation and (iv) general articles on citizen science. Each article was classified under these focus areas using the criteria defined in S1 Table.

The fourth step was to classify the citizen science projects according to the typology developed by Wiggins and Crowston [16], into the five types: action, conservation, investigation, virtual or education, as described in more detail in S1 Table. The coding of each article was performed by a single investigator based on the content of the abstract. A random selection of 60 articles were coded independently by a second investigator to determine the level of agreement and used as a measure of the reliability of the classification process, which showed a discrepancy of ±4% in the coding process.

**Fig 2:**
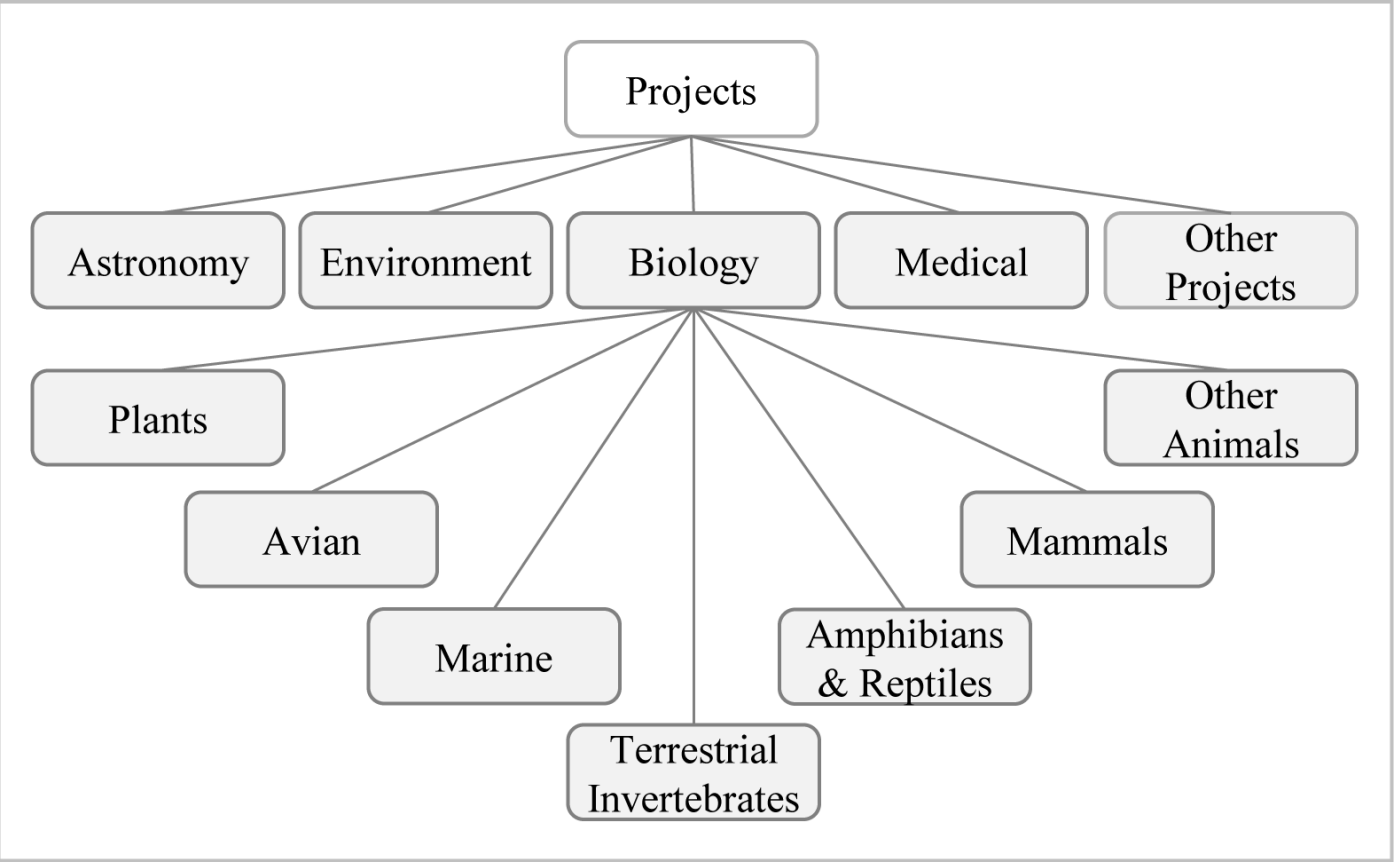
Categorization of citizen science projects into topics.

The fifth step was classification of the citizen science projects into the broad topic areas of astronomy, environment, biology and medical (Fig 2). Topics that were not covered in these headings were placed in the “other” topic category. As the biology component was significant, this category was further divided into the following groups: avian, terrestrial invertebrates, marine organisms, herpetology (amphibians and reptiles), mammals and plants. This grouping deviated from standard animal groups by incorporating marine invertebrates under “marine organisms” as marine projects often incorporated them both. Generic studies of animals such as roadkill studies were classified in the “other animals” category.

In addition, projects that indicated that they used data from past citizen science projects were identified. This was done by tagging articles that specifically mentioned using databases that were obtained by citizen science or public monitoring programs such as the “Christmas Bird Count”.

For each of the above classification types, the percentage and number in each of the categories were calculated and shown against the year of publication to explore changes that occurred over time.

The last step analysed the journals where the articles were published, to determine the most popular journals for the citizen science publications and also the spread of articles over these journals. This was done by creating a list of the unique journal names from the master list, and then counting the number of articles list that appeared in each of these journals for each year of publication.

## Results and Discussions

A total of 1656 articles were extracted, 815 articles from the Web of Science and 841 from Scopus. As 529 articles were duplicated, either appearing in both the Web of Science and Scopus collections or appearing twice in either Web of Science or Scopus collections, 1127 unique articles were identified. After checking the articles against the citizen science definition, this list was reduced to 888 forming the basis for all the following analysis.

**Fig 3:**
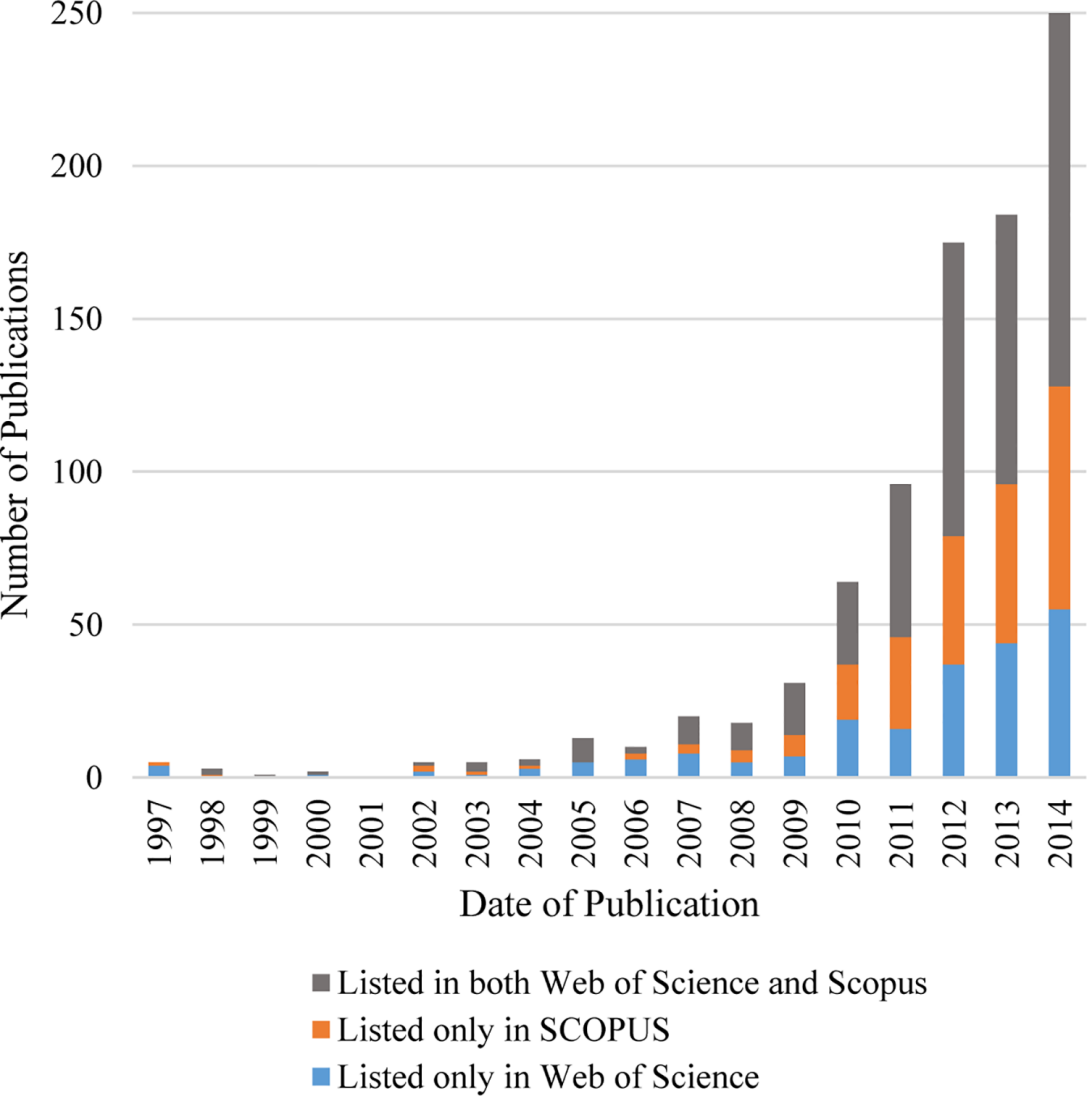
The growth of published peer reviewed articles on citizen science.

The analysis of published dates showed that, though the first publication was in 1997, few publications followed during the next 10 years. In 2007, 6 papers were presented at the Ecological Society of America Meeting, which included general articles and projects on hummingbirds and butterflies and this exposure may have contributed to a substantial increase in publications from that date, as seen in Fig 3. Web of Science accounts for 73% of the extracted articles while Scopus accounts for 76% with 49% of the articles appear in both.

**Fig 4:**
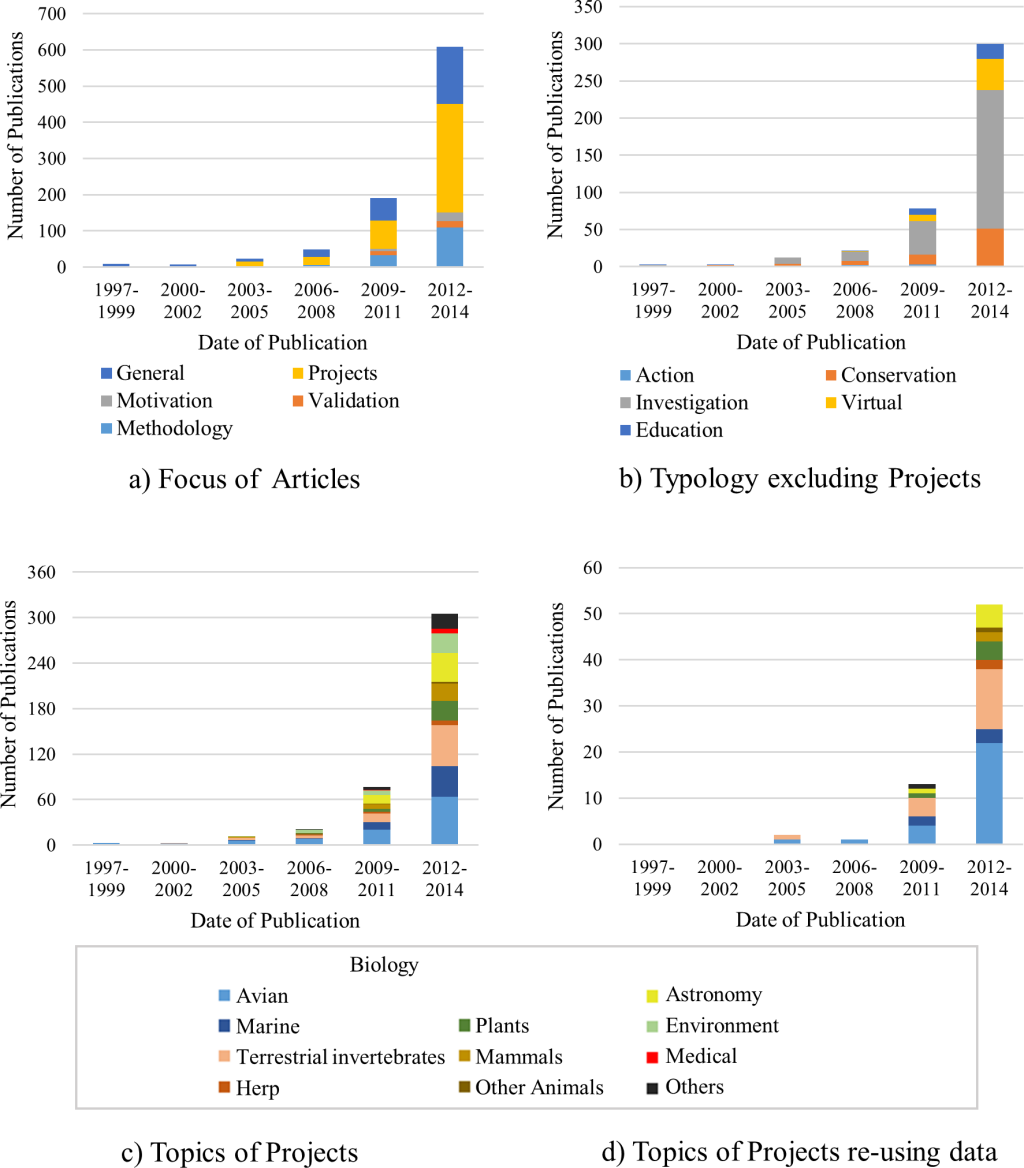
Graphical View of changes in classifications of published articles from 1997 to 2014.

Citizen Science projects were the most common focus for the articles (47%). General articles that contained overviews and reviews of citizen science were also popular (29%) as were as articles discussing methodology (17%). The focus on validation studies was lower (3%) although it should be noted that most of the citizen science projects discussed the method that they used for their project and how their data was validated and some of these articles on projects suggested validation techniques applicable to other projects. The mix of articles has changed over time. Initially all the articles were either general articles on citizen science or specific projects. Articles concentrating on methodology and validation became popular after 2003 as shown in Table 1 and Fig 4A. Studies on the motivation of citizens, and the effect on the citizens are more recent and fewer in number.

**Table 1:** The percentage of published articles divided into focus areas from 1997 to 2014.

Focus Areas	1997–1999	2000–2002	2003–2005	2006–2008	2009–2011	2012–2014	All Years
Methodology	0%	0%	13%	10%	17%	18%	17%
Validation	0%	0%	0%	2%	6%	3%	3%
Motivation / Benefits	0%	0%	0%	0%	3%	4%	3%
Projects	33%	43%	54%	46%	41%	49%	47%
General	67%	57%	33%	42%	33%	26%	29%

The methodology category was found to include articles on tools to facilitate citizen science research, such as usability, the ability to adapt tools for individual use without reprogramming, the incorporation of artificial intelligence techniques to improve both performance and usability, the design electronic guide books to assist and improve classification of species and addressing security and privacy concerns. Publications on methodology also addressed management of large data, including both structured and unstructured data, and methods of combining different datasets to address global issues. Methods used to set up and manage citizen science projects, including the data collection to meet project aims, were also assigned in this category.

The validation category included articles reporting on the effect of training on accuracy, how various characteristics of participants affected their accuracy, aspects of project design on accuracy, and the development of effective frameworks for eliminating bias. This category includes comparisons of participants with experts and the validation of results by multiple observations particularly applicable in virtual projects.

The citizen science projects were divided into their typology group based on their goals, defined as action, conservation, investigation, virtual and education [16]. Only four articles were found to fit into the action category, where the projects are initiated and driven by the public and, as such, did not generally result in scientific publications. The public preferred to publish their outcomes in societal publications, such as newspaper articles, television, presentation, websites and social media, as these sources are more readily available to the wider audience [17, 38]. The investigation category accounted for over half (61%) the articles on projects, as shown in Table 2 and Fig 4B, covering articles focused on scientific discovery in a physical setting. The conservation category was also popular with 18% focusing on physical studies with a resource management, rather than scientific focus. This included, for instance, the road watch study, which recorded road kills in Canada, addressing concerns from both human safety and wildlife conservation perspectives with the aim of mitigating the effects of highway expansion [39]. It should be noted that many of the articles in the investigation category also addressed conservation issues, for example studies which investigated the decline of a particular species with the objective of uncovering the underlying causes for this decline may result in better conservation methods [40]. The main difference between conservation and investigation were that investigations are typically initiated and run by the scientists, and focus on obtaining scientifically valid data for research. With the popularity of Galaxy Zoo resulting in numerous publications, it is surprising that virtual projects comprised only 12% of all the projects. This low fraction may be because citizen science projects did not always cite the origins of their data. For example Galaxy Zoo published a list of 48 publications (up to the end of 2014) based on data obtained by their citizen scientists [41]. Only 4 were contained within the analysed list where the topic is restricted to those referring to “citizen science”. The missing articles discussed discoveries generated using “galaxy zoo” data, rather than acknowledging the contributions of the citizens who created this data. This indicates that the contribution of citizen science to science in general is significantly greater than apparent from literature on citizen science. Virtual projects are likely to grow with recent projects based on using publically available data sources, such as Google Earth which is used for projects such as the discovery of new archaeological sites [42] and publically available picture archives for discovering and tracking species such as the whale-shark [43]. The education category was not significantly represented (7%) and consisted mainly of projects performed in the classroom or school grounds often as part of a science curriculum, such as the butterflies and ground squirrel monitoring projects [21].

**Table 2:** The number of articles on citizen science projects broken into their typologies groups from 1997 to 2014.

Typology	1997–1999	2000–2002	2003–2005	2006–2008	2009–2011	2012–2014	All Years
Action	0	0	0	2	3	0	5
Conservation	0	2	4	6	13	51	76
Investigation	2	0	8	12	45	187	254
Virtual	0	0	0	1	9	42	52
Education	1	1	0	1	8	20	31
Total	3	3	12	22	78	300	418

Biology dominated the topics of citizen science projects, with 72% of the projects in this category (Table 3 and Fig 4C). As well as being the most dominant topic, it has been the area with the most rapid recent growth with the most common objective being to study the diversity and distribution of species [44]. This dominance may be attributed to Cornell University’s Lab of Ornithology laying the foundation for the application of this methodology and targeting the fields of biodiversity monitoring and biological research [4]. The other projects were spread between Astronomy and the Environment as seen in Table 3 and Fig 4C. The “other” category contained diverse topics that do not fit into the previous topics, such as transcribing historical weather records from shipping logs for climate change research [45], disaster recovery and risk assessment [46] and analysing automobile data for monitoring traffic [47]. A new emerging area for citizen science is medical research, such as a project where citizens align multiple sequences of DNA by playing games [48]. The first medical study appeared in the analysed list in 2012.

**Table 3:** The number of citizen science projects for each topic from 1997 to 2014.

Topic	1997–1999	2000–2002	2003–2005	2006–2008	2009–2011	2012–2014	All Years
Astronomy	0	0	0	0	10	34	44
Environment	0	0	1	5	9	37	52
Biology	3	3	11	16	60	234	327
Medical	0	0	0	0	1	8	9
Others	0	0	0	0	4	20	24
Total	3	3	12	21	84	333	456
**Break-down of Biology Category**							
Avian	3	2	6	8	22	70	111
Marine	0	0	1	1	10	42	54
Terrestrial invertebrates	0	1	3	4	13	56	77
Herpetology	0	0	0	1	2	7	10
Plants	0	0	0	1	6	29	36
Mammals	0	0	1	0	6	28	35
Other Animals	0	0	0	1	1	2	4

Birds were the first species recorded in the list [49] and still remain the dominant research topic (24%). Terrestrial invertebrates were the second most common category (18%), with 80% of the studies in this area being on butterflies or moths. The next most popular topic was marine studies (12%), which demonstrated the diverse methods of engaging the public [50]. Observations recorded by recreational divers and fishermen [51] were the basis of 24% of the marine studies, followed by tourism based activities (14%) such as whale watching [52] and intertidal and shallow water studies [53]. Analysis of available images, for example of sharks [43] were also utilized in the marine category. Bats [54] were the most common mammals studied in a diverse category that includes coyotes [55], squirrels [56], otters [19] and koalas [57]. Studies on plants accounted for 7% of the studies.

A number of articles were also focused on multiple species, such as birds and insects, birds and flowers, and squirrels and butterflies. These studies were included in both topics in the above table.

An increasing number of articles reused data from previous citizen science studies [58] for new research objectives. Although citizens may have not been directly involved in these new projects, they could not have been accomplished without the preceding citizen science projects. Birds appeared in the master list as the most common topic in research based on past projects (41%) as seen in Table 4 and Fig 4D. The main driver of this reuse is the freely available data from the eBird project [59] which contains over half a billion records. The eBird web site [60] claimed that over 120 peer-reviewed publications have used their data, and that there have been over 6,500 requests for download in an 18 month period. Only 29 papers appeared in the analysed list which was restricted to those with “citizen science” in their topic, abstract or keywords. The difficulty of discovering papers reusing data from citizen science was highlighted by a recent analysis [61] of research papers used by an article on climate change using avian migration data [62] which found that 85 of the 171 papers referenced in that study were based on citizen science, but the term citizen science never appeared in any of these referenced papers, relying on the researcher’s knowledge of specific program names to identify the source of the data. Terrestrial invertebrates appear on the master list as a topic with the highest percent (24%) of articles based on re-used data and, of these, butterflies were the most common subject. The availability of long term public databases, such as the North America Butterfly Association’s database, which is increasingly being used by scientists to study population trends [63], is an important factor for enabling citizen science data to be re-used.

**Table 4:** The number articles acknowledging the use of citizen science data from past projects grouped by topic from 1997 to 2014.

Topic	1997–1999	2000–2002	2003–2005	2006–2008	2009–2011	2012–2014	Total
Astronomy	0	0	0	0	1	4	5
Environment	0	0	0	0	0	3	3
Biology	0	0	2	1	14	52	69
Medical	0	0	0	0	0	0	0
Others	0	0	0	0	0	1	1
**Total**	**0**	**0**	**2**	**1**	**15**	**60**	**78**
Break-down of Biology Category							
Avian	0	0	1	1	5	25	32
Marine	0	0	0	0	2	4	6
Terrestrial invertebrates	0	0	1	0	4	14	19
Herpetology	0	0	0	0	0	2	2
Plants	0	0	0	0	2	4	6
Mammals	0	0	0	0	0	3	3
Other Animals	0	0	0	0	1	0	1

Climate change research is an example where citizen science data was increasingly used [64], and often combined data from multiple different projects. Aggregating data from diverse datasets requires research both into data quality [65], as well as techniques for combining data from studies collected and generated from diverse datasets [66].

The citizen science articles were scattered over many different publications with the 888 articles analysed here appearing in 479 different publication sources. Seventy percent of these articles appeared in publication sources that have only ever published one or two articles on citizen science. PLOS One (25 articles), Frontiers in Ecology and the Environment (19 articles) and Biological Conservation (14 articles) were the most prolific sources of citizen science articles. A special publication on citizen science in Frontiers in Ecology and the Environment contributed to this journals prominence.

The understanding of the spread of articles encourages researchers to search more broadly for information that they can apply to their own research, and may increase the cross-fertilisation of ideas. The imminent Citizen Science Journal [67] will also provide a focus on citizen science articles.

## Conclusion

The term “citizen science” is increasingly appearing in peer reviewed journals, indicating the wider use and acceptance of this term.

In addition to describing projects, and their outcomes, the number of articles addressing methodology and validation indicated that scientists are addressing the concerns that the data collected or analysed may contain errors resulting from utilizing untrained citizens. These articles discussed the causes, and how to design projects that mitigate against these errors. The research work that used and combined datasets available from previous citizen science projects indicated that, in at least some areas, scientists considered the datasets to be of sufficient quality for future research.

Citizen science articles appeared in a wide range of publications which reflects the range of disciplines that utilize citizen science. The authors expect that this broad analysis will encourage researchers to learn from citizen science research in other disciplines that could enhance their own projects. This is particularly applicable to emerging areas for citizen science, such as medicine.

Citizen Science research also included research into the citizens that participate, why they take part and what benefits that they obtained. The direct involvement of the public in research projects ensures that they are less concerned about the findings and purpose of science as well as exposing them to the scientific process. This has the potential to combat the public scepticism of science when confronted with debates in areas such as climate change. The retention of volunteers is critical to ensuring the on-going long term participation and there is potential to build on the current studies in this area with further research.

With the growth in published output and the ability to learn from past experience, it is expected that research using the citizen science method will further increase and expand to new areas.

## Supporting Information

S1 File Publications from Web of Science. The list of publications with “citizen science” in the topic from the Web of Science as at 1 July 2015.
(CSV)

S2 File Publications from Scopus. The list of Publications with “citizen science” in the topic from Scopus as at 1 July 2015.
(CSV)

S1 Table Criteria used in Coding Articles. A table defining the criteria used for coding the articles into their categories.
(DOCX)
