# Supplementary material for: An Analysis of Citizen Science Based Research: Usage and Publication Patterns
Source: PLoS One. 2015 Nov 23;10(11):e0143687. doi: 10.1371/journal.pone.0143687 (PMC4658079; doi:10.1371/journal.pone.0143687)
Supplement: S1 Table — A table defining the criteria used for coding the articles into their categories. (DOCX) [file pone.0143687.s003.docx]

S1 Table Criteria used in coding articles

| **Code** | **Criteria** |
| --- | --- |
| Citizen Science Project | Describes a specific citizen science study in detail  Describes at least preliminary outcomes of the project |
| Methodology | Describes the theory , procedures and tools developed that can be applied to multiple citizen science projects  May analyse multiple citizen science projects to derive principles that can be utilized in future projects  Does not concentrate primarily on techniques for validation of data, or the motivation of participants  Develops new hardware/software available to be used in future projects  Develops or investigates new ways of designing CS projects |
| Validation | Analyses aspects affecting data quality drawing on multiple projects or databases  Develops new ways of measuring or improving data quality  Evaluates data quality across multiple projects |
| Motivation | Articles that discuss methods of motivating citizens and providing rewards (real or virtual) that are applicable to multiple projects  Articles that look at motivation aspects of a single project with minimal description of the project outcomes  Articles addressing how to influence volunteers to contribute |
| Benefits & Effects on Citizens | Analyses the benefits and other effects on citizens based on multiple projects  Articles addressing changes to citizens as a result of participating, such as scientific literacy with minimal description of the project outcomes. |
| General Article | Articles reviewing the citizen science field  Articles discussing multiple projects in general terms  Articles that do not fit into the above categories |
| Action | As a civic agenda, run and planned by citizens |
| Conservation | Projects engaging citizens to collect data to support conservation objectives, usually in affiliation with larger state or federal agencies, with usually with many stakeholders and are regional in scope |
| Investigation | Projects focused on scientific research goals, using citizens to collect data. These are initiated and run by scientists. |
| Virtual | Projects where the citizens contribute on-line using data supplied to them. |
| Education | Projects with an educational aim or providing learning experience for citizens. |
